# From computers to cultivation: reconceptualizing evolutionary psychology

**DOI:** 10.3389/fpsyg.2014.00867

**Published:** 2014-08-12

**Authors:** Louise Barrett, Thomas V. Pollet, Gert Stulp

**Affiliations:** ^1^Department of Psychology, University of LethbridgeLethbridge, AB, Canada; ^2^Department of Social and Organizational Psychology, VU University AmsterdamAmsterdam, Netherlands; ^3^Department of Population Health, London School of Hygiene and Tropical MedicineLondon, UK

**Keywords:** evolutionary psychology, cognition, cognitive integration, modules, extended mind

## Abstract

Does evolutionary theorizing have a role in psychology? This is a more contentious issue than one might imagine, given that, as evolved creatures, the answer must surely be yes. The contested nature of evolutionary psychology lies not in our status as evolved beings, but in the extent to which evolutionary ideas add value to studies of human behavior, and the rigor with which these ideas are tested. This, in turn, is linked to the framework in which particular evolutionary ideas are situated. While the framing of the current research topic places the brain-as-computer metaphor in opposition to evolutionary psychology, the most prominent school of thought in this field (born out of cognitive psychology, and often known as the Santa Barbara school) is entirely wedded to the computational theory of mind as an explanatory framework. Its unique aspect is to argue that the mind consists of a large number of functionally specialized (i.e., domain-specific) computational mechanisms, or modules (the massive modularity hypothesis). Far from offering an alternative to, or an improvement on, the current perspective, we argue that evolutionary psychology is a mainstream computational theory, and that its arguments for domain-specificity often rest on shaky premises. We then go on to suggest that the various forms of e-cognition (i.e., embodied, embedded, enactive) represent a true alternative to standard computational approaches, with an emphasis on “cognitive integration” or the “extended mind hypothesis” in particular. We feel this offers the most promise for human psychology because it incorporates the social and historical processes that are crucial to human “mind-making” within an evolutionarily informed framework. In addition to linking to other research areas in psychology, this approach is more likely to form productive links to other disciplines within the social sciences, not least by encouraging a healthy pluralism in approach.

## INTRODUCTION

As evolved beings, it is reasonable to assume that evolutionary theory has something to offer the study of human psychology, and the social sciences more generally. The question is: what exactly? This question has been debated ever since Darwin (1871) published the Descent of Man, and we appear no closer to resolution of this issue almost 150 years later. Some maintain that evolutionary theory can revolutionize the social sciences, and hence our understanding of human life, by encompassing both the natural and human sciences within a single unifying framework. Wilson’s (1975) *Sociobiology* was one of the first, and most emphatic, claims to this effect. Meanwhile, others have resisted the idea of unification, viewing it as little more than imperialist over-reaching by natural scientists (e.g., Rose, 2000).

The question posed by this research topic puts a different, more specific, spin on this issue, asking whether an evolutionary approach within psychology provides a successful alternative to current information-processing and representational views of cognition. The broader issue of unification across the natural and social sciences continues to pervade this more narrow debate, however, because certain proponents of the evolutionary approach insist that the incorporation of the social sciences into the natural sciences is the only means to achieve a coherent understanding of human life. As Tooby and Cosmides (2005) state, evolutionary psychology “in the broad sense, … includes the project of reformulating and expanding the social sciences (and medical sciences) in light of the progressive mapping of our species’ evolved architecture” (Tooby and Cosmides, 2005, p. 6).

So, what is our answer to this question? The first point to make clear is that any answer we might offer hinges necessarily on the definition of evolutionary psychology that is used. If one settles on a narrow definition, where evolutionary psychology is equated with the views promoted by the “Santa Barbara School”, headed by Donald Symons, John Tooby, Leda Cosmides, David Buss, and Steven Pinker (referred to here as Evolutionary Psychology or simply as EP), then the answer is a simple “no” (see also: Dunbar and Barrett, 2007). If one opts instead to define an evolutionary approach in the broadest possible terms (i.e., simply as an evolutionarily informed psychology), then the answer becomes a cautious and qualified “yes.”

In what follows, we argue that the primary reason why EP fails as a viable alternative to the standard computational approach is because, in all the important details, it does not differ from this approach. We then go on to suggest that the specific evolutionary arguments in favor of EP, which are used to claim its superiority over other approaches, rest on some rather shaky premises, and cannot be used to rule out alternatives in the way that advocates of EP have supposed. In particular, we deal with arguments relating to the reverse engineering of psychological adaptations, and the logical necessity of domain-specific processes (specifically, arguments relating to the poverty of the stimulus and combinatorial explosion). We then move onto a consideration of recent incarnations of the “massive modularity” hypothesis showing that, while these are not vulnerable to many of the criticisms made against them, it is not clear whether these can, in fact, be characterized as psychological adaptations to past environments. We suggest that, taken together, these arguments weaken the case for EP as the obvious framework for psychology. Finally, we go onto suggest an alternative view of psychological processes, cognitive integration [or the extended mind (EM) hypothesis], that we feel has the potential to improve on the current computational approach; one that is relevant to core areas of psychological research, will promote integration between psychology and other cognate disciplines, but also allow for a healthy pluralism both within psychology and across the social sciences more generally.

## THE COMPUTATIONAL CORE OF EVOLUTIONARY PSYCHOLOGY

The primary reason why Evolutionary Psychology cannot offer a successful alternative to computational-representational theories of mind is because it is a computational-representational theory of the mind. Evolutionary Psychology (e.g., Cosmides, 1989; Tooby and Cosmides, 1992, 2005; Cosmides and Tooby, 1994, 1997) is the marriage of “standard” computational cognitive psychology (as exemplified by Chomsky’s computational linguistics, e.g., Chomsky, 2005) with the adaptationist program in evolutionary biology (e.g., Williams, 1966); a combination that its proponents cast as revolutionary and capable of producing greater insight, not only into human cognitive processes, but also into the very idea of “human nature” itself (Cosmides, 1989; Tooby and Cosmides, 1992, Cosmides and Tooby, 1994, 1997).

The revolutionary promise of incorporating evolutionary theory into psychology can be traced to, among others, Tooby and Cosmides (1992) conceptual paper on the “psychological foundations of culture,” their freely available “primer” on evolutionary psychology (Cosmides and Tooby, 1997), along with Cosmides’s (1989) seminal empirical work on an evolved “cheat-detection” module. Another classic statement of how computational theories benefit from the addition of evolutionary theory is Pinker and Bloom’s (1990) paper on language as an “instinct,” where Chomsky’s innate universal grammar was argued to be a product of natural selection (in contrast to Chomsky’s own views on the matter).

In all these cases, strong claims are made that leave no doubt that “computationalism” forms the foundation of this approach. Cosmides and Tooby (1997), for example, argue that the brain’s evolved function is “information processing” and hence that the brain “is a computer that is made of organic (carbon-based) compounds rather than silicon chips” (paragraph 14), whose circuits have been sculpted by natural selection. More recently, Tooby and Cosmides (2005, p. 16) have stated that “the brain is not just like a computer. It is a computer—that is, a physical system that was designed to process information.” While Pinker (2003, pp. 24–27) argues that: “The computational theory of mind ⋯ is one of the great ideas of intellectual history, for it solves one of the puzzles of the ‘mind-body problem’ ⋯ It says that beliefs and desires are information, incarnated as configurations of symbols ⋯ without the computational theory of mind it is impossible to make sense of the evolution of mind.” Accordingly, hypotheses within EP are predicated on the assumption that the brain really is a computational device (not simply a metaphorical one), and that cognition is, quite literally, a form of information processing. In one sense, then, EP cannot offer an improvement on the computational theory of mind because it is premised on exactly this theory. Any improvement on the current state of play must therefore stem from the way in which evolutionary theory is incorporated into this model.

## THE EVOLVED COMPUTER

The unique spin that EP applies to the computational theory of mind is that our cognitive architecture is organized into a large number of functionally specialized mechanisms, or “modules,” that each performs a specific task (e.g., Tooby and Cosmides, 1992; Cosmides and Tooby, 1997; Barrett and Kurzban, 2006). As these modules are the products of natural selection, they can be considered as “adaptations”, or organs of special design, much like the heart or liver. The function of each module is to solve a recurrent problem encountered by our ancestors in the environment of evolutionary adaptedness (EEA), that is, the period over which humans were subject to evolutionary processes, including those of natural selection (Tooby and Cosmides, 1990; Symons, 1992). The EEA therefore represents the sum total of the selection pressures that give rise to a particular adaptation and cannot, strictly speaking, be identified with a particular time or place (Cosmides and Tooby, 1997). In practice, however, based on the argument that, for most of our evolutionary history, humans lived as hunter–gatherers, the EEA is often operationalized to the Pleistocene habitats of East and Southern Africa (although not to any particular location or specific time within this period).

Unlike the notion of computationalism, which is accepted largely without question in psychology and beyond, the concepts of both “massive modularity” and the EEA have met with a large amount of criticism over the years from social and natural scientists alike, as well as from philosophers (e.g., Lloyd, 1999; Buller and Hardcastle, 2000; Rose and Rose, 2000; Buller, 2005; Bolhuis et al., 2011). In general, critics argue that positing modular psychological adaptations to past environments amounts to little more than “just so” story telling, and lacks adequate standards of proof; an accusation that proponents of EP strongly resist and categorically refute (e.g., Holcomb, 1996; Ketelaar and Ellis, 2000; Confer et al., 2010; Kurzban, 2012). As these arguments and counter-arguments have been covered in detail elsewhere (e.g., Conway and Schaller, 2002; Confer et al., 2010), we will not rehearse them again here. Instead, we deal only with those elements that speak to EP’s success as a novel computational theory of mind, and its ability to improve on the model we have currently.

## CAN WE REVERSE ENGINEER PSYCHOLOGICAL ADAPTATIONS?

Clearly, the success of EP stands or fails by its ability to accurately identify, characterize, and test for psychological adaptations. Within EP, the method of “reverse-engineering” is prominent, and relies heavily on analogies to computational algorithms, functions, inputs, and outputs. In essence, the idea behind reverse-engineering is that one can infer the function of an adaptation from analysis of its form. This involves identifying a problem likely to have been encountered by our ancestors across evolutionary time, and then hypothesizing the kinds of algorithmic “design features” that any psychological adaptation would require in order to solve such a problem. Predictions derived from these hypotheses are then put to the test.

As Gray et al. (2003), among others, have pointed out, such a strategy will work provided all traits are adaptations, that the traits themselves can be easily characterized, and that plausible adaptive hypotheses are hard to come by. Unfortunately, these conditions do not always hold, and identifying adaptations is by no means straightforward. Proponents of EP themselves recognize this problem, acknowledging the existence of both by-products (aspects of the phenotype that are present because they are causally coupled to adaptations) and noise (injected by “stochastic components of evolution”; e.g., Cosmides and Tooby, 1997). Nevertheless, Cosmides and Tooby (1997) argue that, because adaptations are problem-solving machines, it remains possible to identify them “using the same standards of evidence that one would use to recognize a human-made machine: design evidence” (paragraph 65). That is, we are able to identify a machine as a TV rather than a stove by referring to the complex structures that indicate it is good for receiving and transforming electromagnetic waves, and not for cooking food. Thus, if one can show that a phenotypic trait has design features that are complexly specialized for solving an adaptive problem, that these could not have arisen by chance alone, and that their existence is not better explained as the by-product of mechanisms designed to solve some other problem, then one is justified in identifying any such trait as an adaptation (Cosmides and Tooby, 1997).

Although this approach seems entirely reasonable when discussed in these terms, there is ongoing debate as to whether this process is as straightforward as this analysis suggests (particularly with respect to differentiating adaptations from by-products, e.g., Park, 2007). Again, much of this debate turns on the appropriate standard of evidence needed to identify an adaptation, particularly in the case of behavior (see, e.g., Bateson and Laland, 2013). Along with detailed knowledge of the selective environment, it is often argued that evidence for a genetic basis to the trait, along with knowledge of its heritability and its contribution to fitness, are necessary elements in identifying adaptations, not simply the presence of complex, non-random design (see Travis and Reznick, 2009). Defenders of EP counter such arguments by noting, first, that as they are dealing with adaptation, and not current adaptiveness, heritability, and fitness measures are uninformative. By an EP definition, adaptations are traits that have reached fixation. Hence, they should be universal, with a heritability close to zero, and measures of current fitness and the potential for future selection cannot provide any evidence concerning the action of past selection (Symons, 1989, 1990). Second, the argument is made that, given we are willing to accept arguments from design in the case of other species, it is inconsistent and unfair to reject such reasoning in the case of humans. For example, Robert Kurzban, a prominent figure in EP and editor of two main journals in the field, has presented several cogent arguments to this effect in the blog associated with the journal, *Evolutionary Psychology*. In response to a paper presenting the discovery of a “gearing” mechanism in a jumping insect of the genus *Issus*, Kurzban (2013) noted that the authors make a strong claim regarding the evolved function of these interlocking gears (the synchronization of propulsive leg movements). He further noted that that this claim was based on images of the gearing structures alone; there was no reference to the genetic underpinnings or heritability of these structures, nor was there any experimental evidence to establish how the gears work, nor how they contributed to fitness. Kurzban’s (2013) point is: if it is permissible for biologists to reason in this way—and to do so persuasively—then why not evolutionary psychologists? (see also Kurzban, 2011b; for a similar example).

On the one hand, this is an entirely fair point. Other things being equal, if evolutionary psychologists and biologists are arguing for the existence of the same phenomena, namely evolutionary adaptations, then the standards of evidence acceptable to one sub-discipline must also be acceptable when used by the other. On the other hand, the phenomena being compared are not quite equivalent. Insect gears are morphological structures, but psychological adaptations are, according to EP, algorithmic processes. Obviously the latter involve morphology at some level, because “all behavior requires underlying physical structures” (Buss, 1999, p. 11), but it is unclear exactly how the psychological mechanism of, say, cheater detection, maps onto any kind of morphological structure within the brain, not least because of the massive degeneracy of neuronal processes (i.e., where many structurally distinct processes or pathways can produce the same outcome). Prinz et al. (2004), for example, modeled a simple motor circuit of the lobster (the stomatogastric ganglion) and were able to demonstrate that there were over 400,000 ways to produce the same pyloric rhythm. In other words, the activity produced by the network of simulated neurons was virtually indistinguishable in terms of outcome (the pyloric rhythm), but was underpinned by a widely disparate set of underlying mechanisms. As Sporns (2011a,b) has suggested, this implies that degeneracy itself is the organizing principle of the brain, with the system designed to maintain its capacity to solve a specific task in a homeostatic fashion. Put simply, maintaining structural stability does not seem central to brain function, and this in turn makes brain function seem much less computer-like.

This, then, has implications for the proponents of EP, who appear to argue for some kind of stable, functionally specialized circuits, even if only implicitly. In other words, the “function from form” argument as applied to EP raises the question of what exactly underlies a “psychological adaptation” if not a morphological structure that can undergo selection? One way around this is to argue that, in line with Marr’s (1982) computational theory of vision, EP is concerned only with the computational and algorithmic level of analysis, and not the implementation at the physical level (e.g., Buss, 1999). In other words, EP deals with the computational “cognitive architecture” of the human mind and not with the structure of the wet brain. Hence, as long as a reliable and predictable output is produced from a specified set of inputs, EP researchers are justified in referring to the mechanisms that produce this output as a psychological adaptation (whatever these might be).

This seems to raise another problem, however, in that the reliability and stability of the underlying psychological mechanism is only inferred from the reliability of the behavior produced under a given set of circumstances, and does not involve identification of the actual computational mechanism itself. In physical terms, as was evident from the lobster example, when we consider how an organism’s neural circuitry operates in the solving of a task, stability does not seem to be preserved at all, even though virtually indistinguishable network activity is produced as output. If this is true for brains in general and if, as Lehrman (1970) argued, “nature selects for outcomes” and does not particularly care how these are achieved, what has been the target of selection, other than the brain itself? In a sense, one could argue that each specific kind of behavior represents the “modular” component, with a vast number of different neural configurations able to produce it. If so, does this also mean there are a variety of different algorithms as well, and that there is equivalent degeneracy at the algorithmic/representational level? In turn, this raises the issue of whether every possible neural/computational configuration that is capable of producing a given behavior can reasonably be considered a target of selection. Viewed like this, the notion of an “evolved cognitive architecture” comprising specialized circuits devoted to solving a given task serves more as a hypothetical construct used to interpret and make sense of behavioral data, rather than a revealed biological truth. This, of course, does not invalidate the approach—hypothetical constructs are the bread-and-butter of contemporary psychological theorizing—but it does make it difficult to maintain the position that the design argument used to account for stable morphological structures, like insect gears, can be applied equally well to psychological phenomena.

It is important to recognize that our argument is not that there “must be spatial units or chunks of brain tissue that neatly correspond to information-processing units” (Barrett and Kurzban, 2006, p. 641; see also Tooby and Cosmides, 1992). As Barrett and Kurzban (2006) make clear, this does not follow logically, or even contingently, from the argument that there are specialized processing modules; functional networks can be widely distributed across the brain, and not localized to any specific region (Barton, 2007). Rather, we are questioning the logic that equates morphological with psychological structure, given recent neurobiological findings (assuming, of course, that these findings are general to all brains). If neural network structure is both degenerate and highly redundant because the aim is to preserve functional performance in a dynamic environment, and not to form stable representational structures based on inputs received, then it becomes less easy to draw a direct analogy between morphological structures and cognitive “structures.”

The computational metaphor does, however, lend itself to such an analogy, and is perhaps the reason why the structure–function argument seems so powerful from an EP point of view. That is, when the argument is couched in terms of “machinery in the human mind” or “cognitive architecture,” psychological phenomena are more readily conceptualized as stable, physical structures (of some or other kind) that are “visible” to selection. If they are seen instead as temporally and individually variable neuronal configurations that converge on reliable behavioral outputs without any stable circuits, as Prinz et al. (2004) demonstrated in the lobster, a shift of focus occurs, and the brain itself is revealed as the complex adaptation we seek. The capacity to produce frequency-dependent, condition-dependent behavior then becomes the realized expression of the complex adaptation that is the brain, rather than these capacities themselves being seen as distinct adaptations.

This does not end the matter, of course, because we still need to understand how highly active degenerate brain circuits can produce flexible behavior. This is an unresolved empirical issue that cannot be tackled by theoretical speculation alone. Rather, we are simply placing a question mark over the idea that it is possible to identify psychological adaptations at the cognitive level, via behavioral output, without any consideration of how these are physically implemented. Given that, according to EP’s own argument, it is the physical level at which selection must act, and this is what permits an analogy to be drawn with morphological structures, then if brains are less computer-like and representational than we thought, the idea that psychological adaptations can be viewed as stable algorithmic mechanisms that run on the hardware of the brain may also require some re-thinking.

## EVOLVED, LEARNED, AND EVOLVED LEARNING CAPACITIES

Another, more positive, corollary of questioning the premise that the brain is a computer with highly specialized, evolved circuits, is that there is less temptation to distinguish between evolved and learned behaviors in ways that generate a false dichotomy. Although Evolutionary Psychologists do not deny the importance of learning and development - indeed there are some who actively promote a “developmental systems” approach (as we discuss below)—the fundamental assumption that the human cognitive system is adapted to a past environment inevitably results in the debate being framed in terms of evolved versus learned mechanisms. When, for example, the argument is made that humans possess an evolved mating psychology, or an evolved cheater detection mechanism, there is the implicit assumption that these are not learned in the way we ordinarily understand the term, but are more akin to being “acquired” in the way that humans are said to acquire language in a Chomskyan computational framework: we may learn the specifics of our particular language, but this represents a form of “parameter setting,” rather than the formation of a new skill that emerges over time. To be clear, Evolutionary Psychologists recognize that particular kinds of “developmental inputs” are essential for the mechanism to emerge—there is no sense in which psychological modules are argued to be “hard-wired” and impervious to outside input—but there is the denial that these mechanisms reflect the operation of domain-general learning principles being applied in a particular environmental context (Tooby and Cosmides, 1992; Cosmides and Tooby, 1997; Buss, 1999; Barrett and Kurzban, 2006).

In contrast, some researchers take the view that development is more than just “tuning the parameters” of modular capacities via specific inputs, but that development involves dynamic change over time in a highly contingent fashion (e.g., Karmiloff-Smith, 1995, 1998; Smith and Thelen, 2003). In this constructivist view, our ability to engage in certain kinds of reasoning about particular domains of interest, such as cheater detection, emerges through the process of development itself. Hence, these kinds of reasoning are likely to be specific to our time and place and may be very different to the kinds of reasoning performed by our ancestors in both the recent and more distant past. These criticisms are often combined with those mentioned above, namely that the evidence for evolved modular mechanisms is not particularly convincing, and is consistent with alternative explanations for the same data. That is, opponents of modular EP argue that we may learn many of the things that EP attributes to evolved psychological adaptations. In this way, learned mechanisms end up being opposed to those that have evolved.

Such an opposition is, however, false because all learning mechanisms, whether general or domain-specific, have evolved, and therefore what is learned is never independent of evolutionary influences. This is something that both critics and proponents of EP alike recognize, and yet the opposition of evolved versus culturally learned behavior continually arises (e.g., Pinker, 2003). Perhaps this is because the argument is framed in terms of adaptation, when the real issue being addressed by both parties is the degree to which there are constraints on our ability to learn, that is, the degree of plasticity or flexibility shown by our learning mechanisms. Evolutionary Psychologists, in essence, argue simply that all humans converge on a particular suite of mechanisms that once enhanced the fitness of our ancestors, through a process of learning that is heavily guided by certain biological predispositions.

## DOES FLEXIBILITY REQUIRE SPECIFICITY?

This is not to say, however, that humans lack flexibility. Indeed, the argument from EP is precisely that “a brain equipped with a multiplicity of specialized inference engines” will be able to “generate sophisticated behavior that is sensitively tuned to its environment.” (Cosmides and Tooby, 1997, paragraph 42). What it argues against, rather, is the idea that the mind resembles a “blank slate” and that its “evolved architecture consists solely or predominantly of a small number of general purpose mechanisms that are content-independent, and which sail under names such as ‘learning,’ ‘induction,’ ‘intelligence,’ ‘imitation,’ ‘rationality,’ ‘the capacity for culture,’ or simply ‘culture.”’ (Cosmides and Tooby, 1997, paragraph 9). This view is usually characterized as the “standard social science model” (SSSM), where human minds are seen as ‘primarily (or entirely)’ free social constructions” (Cosmides and Tooby, 1997, paragraph 10), such that the social sciences remain disconnected from any natural foundation within evolutionary biology. This is because, under the SSSM, humans are essentially free to learn anything and are thus not constrained by biology or evolutionary history in any way (Cosmides and Tooby, 1997).

Tooby and Cosmides’s (1992) attack on the SSSM is used to clear a space for their own evolutionary theory of the mind. Their argument against the SSSM is wide-ranging, offering a detailed analysis of what they consider to be the abject failure of the social sciences to provide any coherent account of human life and behavior. As we do not have space to consider all their objections in detail (most of which we consider ill-founded), we restrict ourselves here to their dismissal of “blank slate” theories of learning, and the idea that a few domain-general processes cannot suffice to produce the full range of human cognitive capacities.

The first thing to note is that Tooby and Cosmides’s (1992) argument against the SSSM bears a striking resemblance to Chomsky’s (1959) (in)famous dismissal of Skinner’s work. This similarly attempted to undercut the idea of general learning mechanisms and replace it with notions of domain-specific internal structure. This similarity is not surprising, given that Tooby and Cosmides (1992) expressly draw on Chomsky’s logic to make their own argument. What is also interesting, however, is that, like Chomsky (1959), Tooby and Cosmides (1992), and Cosmides and Tooby (1997) simply assert the case against domain-general mechanisms, rather than provide empirical evidence for their position. As such, both Chomsky’s dismissal of radical behaviorism and Evolutionary Psychology’s dismissal of the SSSM amount to “Hegelian arguments.” This is a term coined by Chemero (2009) based on Hegel’s assertion, in the face of contemporary evidence to the contrary, that there simply could not be a planet between Mars and Jupiter (actually an asteroid) because the number of planets in the solar system was necessarily seven, given the logic of his own theoretical framework: an eighth planet was simply impossible, and no evidence was needed to support or refute this statement. In other words, Hegelian arguments are those that rule out certain hypotheses *a priori*, solely through the assertion of particular theoretical assumptions, rather than on the basis of empirical data.

In the case of behaviorism, we have Chomsky’s famous “poverty of the stimulus” argument, which asserted, purely on the basis of “common sense” rather than empirical evidence, that environmental input was too underdetermined, too fragmentary, and too variable to allow any form of associative learning of language to occur. Hence, an innate language organ or “language acquisition device” was argued to fill the gap. Given the alternative was deemed impossible on logical grounds, the language acquisition device was thus accepted by default. The Hegelian nature of this argument is further revealed by the fact that empirical work on language development has shown that statistical learning plays a much larger role than anticipated in language development, and that the stimulus may be much “wealthier” than initially imagined (e.g., Gómez, 2002; Soderstrom and Morgan, 2007; Ray and Heyes, 2011).

Similarly, the argument from EP is that a few domain-general learning mechanisms cannot possibly provide the same flexibility as a multitude of highly specialized mechanisms, each geared to a specific task. Thus, a content-free domain-general cognitive architecture can be ruled out a priori. Instead, the mind is, in Tooby and Cosmides’ (1992) famous analogy, a kind of Swiss Army knife, with a different tool for each job. More recently, the metaphor has been updated by Kurzban (2011a), who uses the iPhone as a metaphor for the human mind, with its multitude of “apps,” each fulfilling a specific function.

Rather than demonstrating empirically that domain-general psychological mechanisms cannot do the job asked of them, this argument is instead supported by reference to functional specialization in other organ systems, like the heart and the liver, where different solutions are needed to solve two different problems: pumping blood and detoxifying poisons. Of course, the brain is also a functionally specialized organ that helps us coordinate and organize behavior in a dynamic, unpredictable world. Using the same logic, this argument is extended further, however, to include the idea that our psychological architecture, which is a product of our functionally specialized brain, should also contain a large number of specialized “mental organs,” or “modules,” because a small number of general-purpose learning mechanisms could not solve the wide variety of adaptive problems that we face; we need different cognitive tools to solve different adaptive problems. Analogies are also drawn with functional localization within the brain: visual areas deal only with visual information, auditory areas deal only with auditory information, and so on.

### THE POVERTY OF THE STIMULUS REVISITED

Cosmides and Tooby (1994) use their own version of Chomsky’s poverty of stimulus argument to support this claim for domain-specificity (see also Frankenhuis and Ploeger, 2007 for further discussion) suggesting that “adaptive courses of action can be neither deduced nor learned by general criteria alone because they depend on statistical relationships between features of the environment, behavior, and fitness that emerge over many generations and are, therefore, not observable during a single lifetime alone.” Thus, general learning mechanisms are ruled out, and modular evolved mechanisms deemed necessary, because these “come equipped with domain-specific procedures, representations or formats prepared to exploit the unobserved” (p. 92).

Using the example of incest avoidance to illustrate this point, Cosmides and Tooby (1994) argue that only natural selection can “detect” the statistical patterns indicating that incest is maladaptive, because “ … it does not work by inference or simulation. It takes the real problem, runs the experiment, and retains those design features that lead to the best available outcome” (p. 93). Frankenhuis and Ploeger (2007), state similarly: “to *learn* that incest is maladaptive, one would have to run a long-term epidemiological study on the effects of in-breeding: produce large numbers of children with various related and unrelated partners and observe which children fare well and which don’t. This is of course unrealistic” (p. 700, emphasis in the original). We can make use of Samuels’ (2002, 2004) definition of “innateness” to clarify matters further. According to Samuels’ (2002, 2004), to call something “innate” is simply to say that it was not acquired by any form of psychological process. Put in these terms, Cosmides and Tooby’s (1994) and Frankenhuis and Ploeger’s (2007) argument is that, because it is not possible to use domain-general psychological mechanisms to learn about the long-term fitness consequences of incest, our knowledge must be innate in just this sense: we avoid mating with close relatives because we have a functionally specialized representational mechanism that acts as a vehicle for domain-specific knowledge about incest, which was acquired by a process of natural selection. Note that domain-specificity of this kind does not automatically imply innateness, as Barrett and Kurzban (2006) and Barrett (2006) make clear. Here, however, the argument does seem to suggest that modules must contain some specific content acquired by the process of natural selection alone, and not by any form of learning, precisely because the latter has been ruled out on a priori grounds.

On the one hand, these statements are entirely correct—a single individual cannot literally observe the long-term fitness consequences of a given behavior. Moreover, there is evidence to suggest that humans do possess a form of incest avoidance mechanism, the Westermarck effect, which results in reduced sexual interest between those raised together as children (Westermarck, 1921; also see Shepher, 1971; Wolf, 1995). On the other hand, it is entirely possible for humans to learn with whom they can and cannot mate, and how this may be linked to poor reproductive outcomes—indeed, people can and do learn about such things all the time, as part of their upbringing, and also as part of their marriage and inheritance systems. Although it is true that many incest taboos do not involve biological incest as such (these are more concerned with wealth concentration within lineages), it is the case that mating and marriage with close relatives is often explicitly forbidden and codified within these systems. Moreover, the precise nature of incest taboos may shift over time and space. Victorian England, for example, was a veritable hotbed of incestuous marriage by today’s standards (Kuper, 2010); indeed, Darwin himself, after famously making a list of the pros and cons of marriage, took his first cousin as his wife.

It is also apparent that, in some cases, shifts in how incestuous unions are defined often relate specifically to the health and well-being of children produced. Durham (2002), for example, discusses the example of incest (or *rual*) among the Nuer cattle herders of Sudan, describing how differing conceptions of the incest taboo exist within the population, such that people obey or resist the taboo depending on their own construal of incest. As a result, some couples become involved in incestuous unions, and may openly challenge the authority of the courts, running off together to live as a family. When these events occur, they are monitored closely by all and if thriving children are produced, the union is considered to be “fruitful” and “divinely blessed.” Hence, in an important sense, such unions are free of *rual* (this is partly because the concept of *rual* refers to the hardships that often result from incest; indeed, it is the *consequences* of incest that are considered morally reprehensible, and *not* the act itself). Via this form of “pragmatic fecundity testing,” the incest taboo shifts over time at both the individual and institutional level, with local laws revised to reflect new concepts of what constitutes an incestuous pairing (Durham, 2002).

This example is presented neither to deny the existence of the Westermarck effect (see Durham, 1991 for a thorough discussion of the evidence for this), nor to dispute that there are certain statistical patterns that are impossible for an individual to learn over the course of its lifetime. Rather it is presented to demonstrate that humans can and do learn about fitness-relevant behaviors within their own lifetimes, and can make adaptive decisions on this basis. Personal knowledge of the outcomes from long-term epidemiological study is not needed necessarily because humans can call on the accumulated stores of inter-generational knowledge residing in, and available from, other members of their community. This can be knowledge that is passed on in folklore, stories, and songs, as well as prohibitions and proscriptions on behavior set down in custom and law. As the Nuer example illustrates, we also form our own ideas about such things, regardless of what we learn from others, possibly because people can, in fact, tap into the “long-term epidemiological study” set up by the evolutionary process a long time ago, and which has been running for many years. It would indeed be impossible to learn the pattern required if each individual had to set up his or her own individual experiment at the point at which they were ready to mate, but people potentially can see the outcomes of the “long term study” in the failed conceptions of others. Furthermore, the Nuer example also makes clear that we are capable of updating our existing knowledge in the light of new evidence. Given that any such learning abilities are themselves evolved, there is no suggestion here that incest taboos are free from any kind of biological influence, and are purely socially constructed. What we are suggesting, however, is that this example undermines the notion that domain-general mechanisms cannot, even in principle, do the job required. We agree that an individual who lives for around 70 years cannot learn the outcome of a process that may take several generations to manifest, but this is a completely different issue from whether an individual can learn that certain kinds of matings are known to have deleterious consequences, and what to do about them. Thus, one cannot use this argument as *a priori* proof that evolved content-rich domain-specific mechanisms are the only possible way that adaptive behavior can be brought about.

In other words, this is not an argument specifically about the mechanisms by which we avoid incest, but a general argument against the strategy used to establish the necessity of evolved domain-specific processes: positing that individuals cannot learn the actual fitness consequences of their actions, as defined within evolutionary biology, does not mean that humans are unable to learn to pick up on more immediate cues that reflect the relative costs and benefits that do accrue within a lifetime (cues that may well be correlated with long-term fitness) and then use these to guide their own behavior and that of their descendants. We suggest it is possible for our knowledge of such matters to be acquired, at least partly, by a psychological process during development. Hence, it is not “innate.” Moreover, even if it could be established that domain-specific innate knowledge was needed in a particular domain (like incest), this does not mean that it can be used as an argument to rule out general learning processes across all adaptive problem domains.

In addition to the above examples, Heyes (2014) has recently presented a review of existing data on infants, all of which were used to argue for rich, domain-specific interpretations of “theory of mind” abilities, and shows that these results can also be accounted for by domain-general processes. Heyes and colleagues also provide their own empirical evidence to suggest that so-called “implicit mentalizing ability” could also equally well be explained by domain-general processes, such as those related to attentional orienting (Santiesteban et al., 2013). In addition, Heyes (2012) has suggested that certain cognitive capacities, which have been argued to be evolved, specialized social learning mechanisms that permit transmission of cultural behaviors, may themselves be culturally-inherited learned skills that draw on domain-general mechanisms.

One point worth noting here is that, if data interpreted as the operation of domain-specific processes can be equally well accounted for by domain-general process, then this has important implications for our earlier discussion of “reverse engineering” and inferring evidence of design, as well as for the necessity of domain-specialization. As Durham (1991) suggested, with respect to the issue of incest taboos: “the influence of culture on human phenotypes will be to produce adaptations that appear as though they could equally well have evolved by natural selection of alternative genotypes … cultural evolution can mimic the most important process in genetic microevolution” (p. 289). Therefore, even if a good case could be made that a cognitive process looks well-designed by selection, an evolved module is not the only possible explanation for the form such a process takes.

### THE PARADOX OF CHOICE?

These demonstrations of the power of domain-general learning are interesting because Tooby and Cosmides (1992) also attempt to rule this out on the basis of “combinatorial explosion,” which they consider to be a knock-down argument. They state that, without some form of structure limiting the range of options open to us, we would become paralyzed by our inability to work through all possible solutions to reach the best one for the task at hand. This again seems to be something of a Hegelian argument, for Tooby and Cosmides (1992) simply assert that “[If] you are limited to emitting only one out of 100 alternative behaviors every successive minute, [then] after the second minute you have 10,000 different behavioral sequences from which to choose, a million by the third minute, a trillion by six minutes” with the result that “The system could not possibly compute the anticipated outcome of each alternative and compare the results, and so must be precluding without complete consideration the overwhelming majority of branching pathways” (p. 102).

This formulation simply assumes that any sequence of behavior needs to be planned ahead of time before being executed, and that an exponential number of decisions *have* to made, whereas it is also possible for behavioral sequences to be organized prospectively, with each step contingent on the previous step, but with no requirement for the whole sequence to be planned in advance. That is, one can imagine a process of Bayesian learning, with an algorithm that is capable of updating its “beliefs.” Relatedly, Tooby and Cosmides (1992) apparently assume that each emission of behavior is an independent event (given the manner in which they calculate probabilities) when, in reality, there is likely to be a large amount of auto-correlation, with the range of possible subsequent behaviors being conditional on those that preceded it.

Finally, Tooby and Cosmides’s (1992) argument assumes that that there is no statistical structure in the environment that could be used to constrain the range of options available (e.g., something akin to the affordances described by Gibson (1966, 1979), and that organisms are thus required to compute all contingencies independently of the environment. May et al. (2006), however, have shown that robotic rat pups, provided with a completely random control architecture (i.e., without any rules at all, whether domain-general or domain-specific), were nevertheless able to produce the distinctive huddling behavior of real rat pups, due to the constraining influence of bodily and environmental structures. That is, rather than having to decide among a trillion different options, according to the logic described above, bodily and environmental structures allow for complex behavior to emerge without any decision-making at all. Thus, there is no reason, in principle, to suppose that humans could not be similarly scaffolded and guided by environmental constraints, in ways that would allow general-learning mechanisms to get a grip and, over time, produce functionally specialized mechanisms that help guide behavior. Indeed, this may also be one reason why human infant learning mechanisms take the form they do, with only a limited capacity at first, so as not to overwhelm the system. As Elman (1993) showed, in his classic paper on infant language learning, the training of a neural network succeeded only when such networks were endowed with a limited working memory, and then gradually “matured.” More recently, Pfeifer and Bongard (2007) have reported on similar findings relating to the development of behavior in a “babybot.”

Thus, while reasonable when taken at face value, many of the arguments offered in support of an evolved domain-specific computational architecture turn out to be rather Hegelian on closer inspection, rather than well-supported by empirical data. As such, the increased value of evolutionary psychology remains an open issue: it is not clear that EP offers an improvement over other computational perspectives that do not make strong claims for an evolved, domain-specific architecture of this kind.

## MODULES 2.0

The contention that EP has sometimes offered Hegelian arguments should not be taken to suggest that opponents of the EP position are not guilty of the same. We do not deny that modular accounts have also been ruled out based on assertion rather than evidence, and that there have been many simplistic straw man arguments about genetic determinism and reductionism. Interestingly enough, Jerry Fodor himself, author of “The Modularity of Mind” (Fodor, 1983), asserted that it was simply impossible for “central” cognitive processes to be modular, and Fodor (2000) also presents several Hegelian arguments against the evolutionary “massive modularity” hypothesis. Indeed, the prevalence of such arguments in the field of cognitive science is Chemero’s (2009) main reason for raising the issue. His suggestion is that, unlike older disciplines, cognitive science gives greater credence to Hegelian arguments because it has yet to establish a theoretical framework and a supporting body of data that everyone can agree is valid. This means that EP does not present us with the knock-down arguments against the SSSM and domain-general learning that it supposes, but neither should we give Hegelian arguments against EP any credence for the same reason.

As both Barrett and Kurzban (2006) and Frankenhuis and Ploeger (2007) have documented, many of the misrepresentations and errors of reasoning concerning the massive modularity hypothesis in EP can, for the most part, be traced precisely to the conflation of Fodor’s (1983) more limited conception of modularity with that of Tooby and Cosmides (1992, 2005) and Cosmides and Tooby (1994). Criticisms relating to encapsulation, cognitive impenetrability, automaticity, and neural localization are not fatal to the EP notion of modularity because EP’s claim is grounded in functional specialization, and not any specific Fodorian criterion; criticisms that argue in these terms therefore miss their mark (Barrett and Kurzban, 2006).

Given that most criticisms of the massive modularity hypothesis prove groundless from an EP point of view, it is worth considering Barrett and Kurzban’s (2006) analysis in detail in order to understand exactly what the EP view of modularity entails, and whether this updated version of the modularity argument is more convincing in terms of presenting an improved alternative to standard computational models.

First and foremost, Barrett and Kurzban (2006) make clear that functional specialization *alone* is the key to understanding modularity from an EP point of view, and domain-specific abilities, and hence modules, “should be construed in terms of the formal properties of information that render it processable by some computational procedure” (Barrett and Kurzban, 2006, p. 634). That is, modules are defined by their specialized input criteria and their ability to handle information in specialized ways: only information of certain types can be processed by the mechanism in question. Natural selection’s role is then “to shape a module’s input criteria so that it processes inputs from the proper domain in a reliable, systematic and specialized fashion.” (By “proper” domain they mean the adaptive problem, with its associated array of inputs, that the module has been designed by selection to solve; this stands in contrast to the “actual” domain, which includes the range of inputs to which the module is potentially able to respond, regardless of whether these were present ancestrally: see Sperber, 1994; Barrett and Kurzban, 2006, p. 635). Hence, the domain-specificity of a module is a natural consequence of its functional specialization (Barrett and Kurzban, 2006). Crudely speaking, then, modules are defined more in terms of their syntactic rather than semantic properties—they are not “content domains,” but more like processing rules.

Barrett and Kurzban (2006) argue that their refinement of the modularity concept holds two implications. First, given that a module is defined as any process for which it is possible to formally specify input criteria, there is no sharp dividing line between domain-specific and domain-general processes, because the latter can also be defined in terms of formally specified input criteria. The second, related implication is that certain processes, like working memory, which are usually regarded as domain-general (i.e., can process information from a wide variety of domains, such as flowers, sports, animals, furniture, social rituals), can also be considered as modular because they are thought to contain subsystems with highly specific representational formats and a sensitivity only to specific inputs (e.g., the phonological loop, the visuospatial sketchpad; Barrett and Kurzban, 2006). This does, however, seem to deviate slightly from Cosmides and Tooby’s (1997) suggestion that modules are designed to solve particular adaptive problems encountered by our ancestors: what *specific* adaptive problem does “working memory” solve, given that the integration of information seems common to all adaptive problems? (see also Chiappe and Gardner, 2012).

Taken on its own terms, however, Barrett and Kurzban’s (2006) definition of modularity should raise no objections from anyone committed to the computational theory of mind, nor does it come across as particularly radical with respect to its evolutionary theorizing. Thus, Barrett and Kurzban (2006) dissolve many of the problems identified with massive modularity, and suggest that most criticisms are either misunderstandings or caricatures of the EP position. When considered purely as a computational theory (i.e., leaving to one side issues relating to the EEA, and Hegelian arguments relating to the need for evolved domain-specific knowledge), the more recent EP position is thereby revealed as both reasonable and theoretically sophisticated.

### DEVELOPMENTAL CONSIDERATIONS: “SOFT” DEVELOPMENTAL SYSTEMS THEORY AND EP

It is also important to note that the more recent work in EP also incorporates a strongly developmental perspective, again laying rest to criticisms that EP is overly determinist and that EP researchers are prone to simplistic claims about the innateness or “hard-wiring” of particular traits (e.g., Barrett, 2006; Frankenhuis et al., 2013). In particular, work by Barrett (2006) and Frankenhuis et al. (2013) attempts to integrate developmental systems theory (DST) into EP. This represents an encouraging move at first glance, because the aim of DST is to moves us away from a dichotomous account of development, where two classes of resources—genes and “all the rest”—interact to produce the adult phenotype, toward an account in which there is no division into two fundamentally different kinds of resources. Instead, genes are seen as just one resource among many available to the developmental process, and are not the central drivers of the process (Griffiths and Gray, 1994). Indeed, genes can play their role only if all other resources essential for development are in place. This should not be taken to mean that all resources contribute equally to each and every process, and always assume the same relative importance: the aim is not to “homogenize” the process of development, and obliterate the distinctions between different kinds of resources, but to call into question the way in which we divide up and classify developmental resources, opening up new ways to study such processes.

The EP take on DST, however, is self-confessedly “soft,” and continues to maintain that standard distinction between genetic and environmental resources. As defined by Frankenhuis et al. (2013), “soft DST” regards developmental systems as “dynamic entities comprising genetic, molecular, and cellular interactions at multiple levels, which are shaped by their external environments, but distinct from them” (p. 585). Although a strongly interactionist view, the “developmental system” here remains confined to the organism alone, and it continues to treat genetic influences as fundamentally distinct from other developmental resources, with a unique role in controlling development. More pertinently, Barrett (2006) suggests that, precisely because it gets us away from any kind of “genetic blueprint” model of growth and development, it may be “fruitful to think of developmental processes themselves in computational terms: they are designed to take inputs, which include the state of the organism and its internal and external environments as a dynamically changing set of parameters, and generate outputs, which are the phenotype, the end-product of development. One can think of this end-product, the phenotype, as the developmental target” (p. 205). Thus, once again, EP does not present us with an alternative to current computational models, because, as Barrett (2006) makes clear, the incorporation of these additional theories and models into an EP account entails a reinterpretation of such theories in fully computational terms.

### ANCIENT ADAPTATIONS OR THOROUGHLY MODERN MODULES?

Another consideration we would like to raise is whether, as a result of incorporating a clearly articulated developmental component, EP researchers actually undermine some of their own claims regarding the evolved domain-specificity of our putative modular architecture. Barrett (2006), for example, uses Sperber’s (1994) ideas of actual and proper domains to good effect in his developmental theorizing, distinguishing clearly between “types” of cognitive processes (which have been the target of selection) and “tokens” of these types (which represent the particular manner in which this manifests under a given set of conditions). This enables him to provide a cogent account of an evolved modular architecture that is capable of generating both novelty and flexibility. The interesting question, from our perspective, is whether the modules so produced can be still be considered adaptations to past environments, as Cosmides and Tooby (1994, 1997) insist must be the case.

For example, as Barrett (2006) notes, many children possess the concept of *Tyrannosaurus rex*, which we know must be evolutionarily novel because, as a matter of empirical fact, there has been no selection on humans to acquire this concept. Nevertheless, as Barrett (2006) argues, we can consider the possession of this concept as a token outcome that falls well within the proper type of a putative predator-recognition system. This argument is logical, sensible, and difficult to argue with, yet seems at odds with the central idea presented in much of Tooby and Cosmides (1992, 2005) work that the modular architecture of our minds is adapted to a past that no longer exists. That is, as tokens of a particular type of functional specialization, produced by a developmental process that incorporates evolutionarily novel inputs, it would seem that any such modules produced are, in fact, attuned to present conditions, and not to an ancestral past. As Barrett (2006) notes, Inuit children acquire the concept of a polar bear, whereas Shuar children acquire the concept of a jaguar, even though neither of these specific animals formed part of the ancestral EEA; while the mechanisms by which these concepts are formed, and why these concepts are formed more easily than others, may well have an evolutionary origin, the actual functional specializations produced - the actual tokens produced within this proper type - would seem to be fully modern. The notion that “our modern skulls house a stone age mind” (Cosmides and Tooby, 1997) or that, as Pinker (2003, p. 42) puts it; “our brains are not wired to cope with anonymous crowds, schooling, written language, governments, police courts, armies, modern medicine, formal social institutions, high technology and other newcomers to the human experience” are thus undermined by the token-type distinction developed in more recent EP theorizing.

One could argue, perhaps, that what Pinker means here is that our brains did not evolve to deal with such things specifically, i.e., that he is simply making Barrett’s (2006) argument that these phenomena are just tokens of the various types that our brains *are* wired to cope with. But, if this is the case, then it seems that EP loses much of its claim to novelty. If it is arguing only that humans have evolved psychological mechanisms that develop in ways that attune them to their environment, this does not differ radically from computational cognitive theories in human developmental and comparative psychology more generally.

This sounds like a critical argument, but we do not mean it in quite the way it sounds. Our argument is that the theoretical EP literature presents a perfectly acceptable, entirely conventional computational theory, one that admits to novelty, flexibility, the importance of learning and development, and incorporates the idea that a species’ evolutionary history is important in shaping the kinds of psychological processes it possesses and the ease with which they are acquired. Our point is that this is no different from the arguments and empirical findings offered against behaviorism toward the middle of the last century, which heralded the rise of cognitive psychology (see e.g., Malone, 2009; Barrett, 2012). Central to all cognitivist psychological theories is the idea that there are internal, brain-based entities and processes that transform sensory input into motor output, and the acknowledgment that much of this internal structure must reflect a past history of selection. EP, in this sense then, is not controversial within psychology, and is entirely consonant with current psychological theory and practice. Thus, in addition to the fact that EP is based on the same computational metaphor as standard cognitive psychology, it is also apparent that most of the evolutionary aspects of this theory, as reconceived by current authors, do not render it revolutionary within psychology, nor is there any reason to believe that the remaining social sciences should view EP as any more essential or necessary to their work than current computational models. Indeed, one could simply take the message of EP to be that, as with all species, humans are prepared to learn some things more readily than others as a result of evolving within a particular ecological niche. Seen in these terms, it is surprising that EP continues to be considered controversial within psychology, given that its more recent theoretical claims can be seen as entirely mainstream.

## AN ALTERNATIVE SUGGESTION: COGNITIVE INTEGRATION

If our conclusion is that EP does not offer an alternative to standard computational cognitive psychology, we are left with two further questions: Is an alternative really needed? And if so, what is it? In the remainder of this paper, we tackle these questions in turn.

One reason why we might need an alternative to standard computational and representational theories of mind is because, despite claims to the contrary (e.g., Pinker, 2003), it has yet to provide a complete account of how humans and other species produce adaptive, flexible behavior in a dynamic, unpredictable world. Although we may understand something about capacities like playing chess, engaging in formal reasoning, or natural language (i.e., tasks that involve the manipulation of symbols according to rules, and are inherently computational anyway), we still lack a good understanding of the more mundane tasks that characterize much of what we could call “everyday” intelligence, such as how we manage to negotiate uneven terrain or coordinate all the actions and objects necessary to make a pot of tea, or coordinate our social actions with others when we dance, engage in conversation smoothly and easily, or simply walk down a crowded street.

It is also interesting to note that the computational metaphor also hindered the advancement of robotics in much the same way. The MIT roboticist and inventor of the Roomba, Rodney Brooks, relates how his first formal foray into robotics was at Stanford, where they took a “classic” artificial intelligence approach, with robots that took in sensory inputs, computed solutions to a task based on these inputs, and then executed them. This made the robots operate very slowly, even to the extent that the movement of the sun across the sky, and the changes in the shadows thrown, had the ability to confuse their internal representations. Only by moving away from a classic computational “sense–represent–plan–act” approach, and eliminating the need for internal representations altogether, was progress made (Brooks, 2002; also see Pfeifer and Bongard, 2007).

In other words, the idea that cognition is, ultimately, a form of “mental gymnastics” (Chemero, 2009) involving the construction, manipulation, and use of internal representations according to a set of rules does not seem to provide an adequate account of how humans and other animals achieve most of the activities they engage in every day. Given this, the obvious alternatives to the standard computational theories of mind are the various forms of “E-cognition” (embodied, embedded, enactive, extended, and extensive) that have been gaining steady ground in recent years within cognitive science and philosophy of mind and, to a lesser extent, psychology itself, both theoretically and empirically (e.g., Clark, 1997, 2008; Gallagher, 2005; Wheeler, 2005; Menary, 2007, 2010; Pfeifer and Bongard, 2007; Chemero, 2009; Barrett, 2011; Hutto and Myin, 2013). While these approaches vary in the degree to which they reject computational and representational approaches to cognition [e.g., Clark (1997, 2008) argues for a form of “dynamic computationalism,” whereas Hutto and Myin (2013) reject any suggestion that “basic minds,” i.e., those that are non-linguistic, make use of representational content], they have in common the idea that body and environment contribute to cognitive processes in a constitutive and not merely causal way; that is, they argue that an organism’s cognitive system extends beyond the brain to encompass other bodily structures and processes, and can also exploit statistical regularities and structure in the environment.

For reasons of space, we cannot provide a full account of these alternatives, and the similarities and differences between them. Instead, we will focus on one particular form of E-cognition, the “EM” hypothesis. Specifically, we will deal with “second-wave EM” thinking, also known as “cognitive integration,” as exemplified by the work of Clark (2008), Sutton (2010), and Menary (2007, 2010). We believe this supplies the beginnings of an answer to why an alternative to standard computational theory is required, and illustrates why EP cannot provide it.

Put simply, the EM hypothesis is that external resources and artifacts, like written language and other forms of material culture, are central to the production of the modern human cognitive phenotype, and serve to augment and ratchet up the power of our evolved brains (e.g., Clark, 1997, 2008; Menary, 2007, 2010; Sutton, 2010). External resources are argued to play a role in a cognitive process in ways that are either functionally equivalent to that carried out by a biological brain such that, for the duration of that process, the external resource can be considered to be part of the cognitive system (the so-called “parity principle”: Clark and Chalmers, 1998) or they play roles that are complementary to brain-based processes, and augment them accordingly (the “complementarity principle”: Menary, 2007, 2010; Sutton, 2010). We can see this in everything from the way in which our ability to multiply very large numbers is enhanced by the use of pencil and paper to the fascinating literature on sensory substitution devices, where blind individuals are able to visually explore their environments via external devices that supply auditory or tactile information in ways that compensate for the loss of their visual sense (Bach-y-Rita et al., 1969, 2003; Bach-y-Rita and Kercel, 2003). The idea here, then, is not to eliminate all distinctions between different kinds of resources and consider them to be synonymous, but to reduce our prejudice that only internal processes taking place in the brain count as cognitive, and to redraw the boundaries of the cognitive system accordingly. The notion of the EM or cognitive integration therefore dissolves the boundary between brain, body, and world, and rejects the idea that the “cognitive system” of an animal is confined to its brain alone (for a review of how cognitive integration relates to the non-human animal literature, see Barrett, 2011). Instead, as Clark (1997) and Clark and Chalmers (1998) suggested, many of our cognitive states can be considered as hybrids, distributed across biological and non-biological realms. We are, as the title of one of Clark’s books suggests, “natural born cyborgs” (Clark, 2003).

### CULTIVATING THE HYBRID HUMAN

The human cognitive system, in particular, is extended far beyond that of other species because of the complex interaction between the biological brain and body, and the wide variety of artifacts, media and technology that we create, manipulate, and use. It is crucial to realize that the hybrid nature of human beings is not a recent phenomenon tied to the development of modern technology. On the contrary, cognitive extension is a process that has been taking place ever since the first hominin crafted the first stone tools, and has continued apace ever since. What this means today is that, as Clark (2003) puts it, “our technologically enhanced minds are barely, if at all, tethered to the ancestral realm” (p. 197) nor are they now “constrained by the limits of the on-board apparatus that once fitted us to the good old savannah” (p. 242). This stands in stark contrast to the EP position, where the only “cognitive machinery” involved is the brain itself, whose structure is tied fundamentally and necessarily to the past, untouched by our culturally constructed, technological world. As Tooby and Cosmides (1992) put it: “what mostly remains, once you have removed from the human world everything internal to humans, is the air between them” (p. 47). Cognitive integration begs to differ in this regard, and invites us to look around and see that this simply cannot be true.

Consequently, our view is that cognitive integration promises to explain more about human psychology than EP ever could because it forces a stronger recognition of the historical, sociocultural nature of human psychology - the fact that we develop in a socially and culturally rich milieu that reflects the contingent nature of both historical and evolutionary events. Past generations structure the developmental context of those that succeed them, providing resources that are essential to the production of species-typical behavior. Importantly, however, they also enhance what can be achieved by providing ever more sophisticated forms of cognitive scaffolding that itself augments the scaffolding that previous generations bequeathed to them (Sterelny, 2003; Stotz, 2010). This can be seen as something akin to the process of ecological succession, where the engine of change is the organism’s own impact on the environment; a metaphor we have stolen from Griffiths and Gray’s (1994) treatment of DST. Indeed, there is a natural sympathy between DST as an approach to the study of the evolution and development of biological organisms, and the more dynamical forms of E-cognition that adopt a similar approach to the evolution, development, and functioning of cognitive systems. In particular, Stotz (2010) argues convincingly that understanding human psychology from an evolutionary perspective requires a focus on “developmental niche construction”; an idea that, as the name suggests, incorporates elements of both developmental systems and niche construction theory (see also Griffiths and Stotz, 2000). Understanding modern human psychology therefore requires an understanding of the entanglement of our technologies, cultural practices, and historical events with our evolutionary heritage, and not the reverse engineering of human cognitive architecture alone. Clark (2002) suggests that the pay-off from this kind of expanded psychology “… could be spectacular: nothing less than a new kind of cognitive scientific collaboration involving neuroscience, physiology and social, cultural and technological studies in equal measure” (p. 154).

Turning to an embodied, extended approach as an alternative to standard computational theories, including that of EP, is a step in the right direction not only because it recognizes the hybrid nature of humans, in the terms described above, but also in the sense discussed by Derksen (2005), who argues that a recognition of ourselves as part-nature and part-culture creates a distinct and interesting boundary (or rather a range of related boundaries) between humans and the natural world. As Derksen (2005) points out, the reflexive ways in which we deal with ourselves and our culture are very different from our dealings with the natural world, and a recognition of our hybrid nature allows us to explore these boundaries in their own right, and to examine how and why these may shift over time (for example, issues relating to fertility treatments, stem cell research, cloning, and organ transplantation all raise issues concerning what is “natural” versus “unnatural,” and how we should conceive of human bodies in both moral and ethical terms).

To emphasize this shifting, dynamic element of the boundaries we straddle as hybrid natural-cultural beings, Derksen (2005) uses the metaphor of “cultivation.” Like a gardener tending his plants, humans cultivate their nature, and in so doing elaborate their potential. As Vygotsky (1962) suggested, this makes culture something we do, rather than something that happens to us, or that we simply possess. The intersection between cognitive integration and cultivation should be clear, for cognitive integration, which naturally takes into account our historical, social, cultural, and evolutionary underpinnings in equal measure, is key to our ability to cultivate new forms of human nature (see also Bakhurst, 2011). Indeed, proponents of cognitive integration, suggest that “human nature” continually emerges in an ongoing way from human activity, and that we cannot pinpoint some fixed and unchanging essence (Derksen, 2012). As Wheeler and Clark (2008) put it: “our fixed nature is a kind of meta-nature … an extended cognitive architecture whose constancy lies mainly in its continual openness to change” (p. 3572).

Such a view stands in contrast to the EP perspective, where the idea of a universal human nature, comprising our evolved computational architecture, is a central premise of the approach. The problem here, as we see it, is that cultural variation across time and space is seen simply as the icing on the cake of our evolved universal psychology. Humans are argued to manifest different behaviors under different conditions because our evolved architecture works rather like a jukebox that can play different records given different inputs; what Tooby and Cosmides (1992) refer to as “evoked culture.” By this definition, such cultural differences fail to penetrate or alter our “human nature” in any fundamental way. Such a view also fails to account for how and why completely different modes of thinking have emerged over space and time as a consequence of the invention of different material artifacts, like the wheel, the plow, time-pieces, accounting systems, and written language. Such things are not evoked simply by exposure to local ecological conditions, and their existence fundamentally changes how we think about the world (without the invention of time-pieces, for example, the cultural importance of timeliness and punctuality so valued by, among others, the Swiss and Germans, would not, and could not, be considered any part of human nature). EP therefore leaves out the most distinctive aspect of human cognitive life—the way in which material culture is both a cause and consequence of our psychological and cultural variability—whereas cognitive integration makes this the central element to understanding why humans think and act in the ways that they do (Menary, 2010; Sutton, 2010; Malafouris, 2013).

Finally, as Derksen (2005, 2007) argues, a view of human nature as a matter of cultivation, as a form of ongoing human activity, renders the idea of unification between the biological and social sciences wrongheaded on its face: the very diversity of disciplines in which we engage reflects the disunity, the boundary between nature and culture, that characterizes our humanity, and not the fundamental “psychic unity” of humankind that EP assumes. Consequently, there is a very real need to collaborate and confront each other along disciplinary boundaries, but not dissolve, ignore, or erase them (Derksen, 2005, 2007). Such sentiments are echoed by those involved in the study of cognitive integration, who similarly call for this kind of multidisciplinary pluralism in our approach to the study of human nature and the mind (Derksen, 2005, 2007; Menary, 2007; Clark, 2008; Wheeler and Clark, 2008; Menary, 2010; Sutton, 2010). Simply put, our hybrid selves can be studied in no other way.

## Conflict of Interest Statement

The authors declare that the research was conducted in the absence of any commercial or financial relationships that could be construed as a potential conflict of interest.
